# Differential expression of miRNA-146a and miRNA-155 in gastritis induced by *Helicobacter pylori* infection in paediatric patients, adults, and an animal model

**DOI:** 10.1186/s12879-018-3368-2

**Published:** 2018-09-15

**Authors:** Ana Caren Cortés-Márquez, Sandra Mendoza-Elizalde, Francisco Arenas-Huertero, Jimena Trillo-Tinoco, Pedro Valencia-Mayoral, Alejandra Consuelo-Sánchez, Jonathan Zarate-Franco, Ada Ruth Dionicio-Avendaño, José de Jesús Herrera-Esquivel, Elio Germán Recinos-Carrera, Christian Colín-Valverde, Sandra Rivera-Gutiérrez, Alfonso Reyes-López, Juan Carlos Vigueras-Galindo, Norma Velázquez-Guadarrama

**Affiliations:** 1 Infectology Laboratory, Hospital Infantil de México Federico Gómez, México City, Mexico; 2 Biomedicine and Molecular Biotechnology, Escuela Nacional de Ciencias Biológicas, Instituto Politécnico Nacional, México City, Mexico; 3 Molecular Microbiology Laboratory, Microbiology Department, Escuela Nacional de Ciencias Biológicas, Instituto Politécnico Nacional, México City, Mexico; 4 Laboratory of Research in Experimental Pathology, Hospital Infantil de México Federico Gómez, México City, Mexico; 5 Department of Immunology, Moffitt Cancer Center, Tampa, FL USA; 6 Planning Direction, Hospital Infantil de México Federico Gómez, México City, Mexico; 7 Gastroenterology and Nutrition Department, Hospital Infantil de México Federico Gómez, México City, Mexico; 8 Health Science, Escuela Superior de Medicina, Instituto Politécnico Nacional, México City, Mexico; 9 Endoscopy Department, Hopital General Dr. Manuel Gea González, México City, Mexico; 10 Division of Pathological Anatomy, Hospital General Dr. Manuel Gea González, México City, Mexico; 11 Center of Economic and Social Studies in Health, Hospital Infantil de México Federico Gómez, México City, Mexico

**Keywords:** *Helicobacter pylori*, Gastritis, miRNA-146a, miRNA-155, *Meriones unguiculatus*

## Abstract

**Background:**

*Helicobacter pylori* is a major aetiologic agent associated with gastritis. *H. pylori* infections increase the expression of the Toll-like receptor (TLR), which in turn modulates the expression of microRNA (miRNA)-146a and miRNA-155. The objective of this study was to compare the expression of miRNA-146a and miRNA-155 in gastric lesions of paediatric and adult patients with different pathologies and in Mongolian gerbils (*Meriones unguiculatus*) infected with *H. pylori* 26,695.

**Methods:**

Quantification of miRNA expression was performed by quantitative real-time polymerase chain reaction (qRT-PCR) of paraffin-embedded gastric lesions of children with or without an infection (*n* = 25), adults with follicular gastritis and metaplasia (*n* = 32) and eight-week-old *M. unguiculatus* males (Hsd:MON) infected with *H. pylori* 26,695 for 0, 3, 6, 12 and 18 months (n = 25). The genes RNU48 and RNU6 were used as endogenous controls for data normalization. Statistical analyses were performed using Kruskal-Wallis, Mann-Whitney, ANOVA and Student’s t-test.

**Results:**

The expression of miRNA-146a and miRNA-155 in infected children increased by 247.6- and 79.4-fold (on average), respectively, compared to that observed in the control group. However, these results were not significant (*p* = 0.12 and *p* = 0.07 respectively). In some children a gradual increase in expression was observed, while in others, expression was very high. Additionally, the expression levels of miRNA-146a and miRNA-155 increased by an average of 21.7- and 62-fold, respectively, in adult patients with follicular gastritis when compared to those of the controls. In *M. unguiculatus* infected with *H. pylori* 26,695, the expression of both miRNAs increased as the infection progressed.

**Conclusion:**

This is the first report to show differences in the expression of miRNA-146a and miRNA-155 in paediatric and adult patients with gastritis who were infected with *H. pylori*. In addition, in *M. unguiculatus* infected with *H. pylori*, miRNA expression was associated with the progression of infection and the ability of the bacteria to adapt to the host.

**Electronic supplementary material:**

The online version of this article (10.1186/s12879-018-3368-2) contains supplementary material, which is available to authorized users.

## Background

*Helicobacter pylori* is one of the primary etiological agents associated with gastritis, a condition characterized by inflammation of the lining of the stomach that is associated with mucosal lesions [1]. The progression of *H. pylori* infections is highly variable, and depending on the persistence of inflammation, it can lead to the development of acute gastritis or chronic active gastritis [1].

Patients infected with *H. pylori* develop acute gastritis that may resolve, a type of gastritis is associated with the development of hypochlorhydria and neutrophil infiltration. However, acute gastritis may also develop into chronic active gastritis, which is characterized by the infiltration of mononuclear cells, dominated by lymphocytes, plasma cells and macrophages. Additionally, acute gastritis may lead to multifocal chronic gastritis, for which there are many causes (genetics, age of acquisition, and virulence of the bacterial strain), and patients are frequently asymptomatic. In some individuals, multifocal chronic gastritis induces gastric atrophy and intestinal metaplasia, the latter of which is considered a risk factor for the development of dysplasia, which ultimately results in gastric cancer [1, 2].

microRNAs (miRNAs) are endogenous, non-coding RNAs that are 18 to 24 nucleotides in length and regulate post-transcriptional gene expression in animals and plants [3, 4] by participating in the regulation of various biological processes [3, 5–8]. The expression of many miRNAs are altered in numerous human diseases, such as cancer and immune and inflammatory disorders [4, 9]. In addition, they play important roles in host-parasite regulation, altering the host response to infection and the limiting the dissemination or replication of microorganisms. Specifically, in various infectious diseases, miRNAs regulate the host innate immune response [10, 11]. Furthermore, to facilitate replication, microorganism may directly alter miRNA expression [12]. Matsushima et al. (2011) identified 31 miRNAs exhibiting differential expression in patients with *H. pylori*-infected gastric mucosa [8].

In adults, miRNA-146a and miRNA-155 are specifically involved in negatively regulating the pro-inflammatory immune response and are transcriptionally induced during *H. pylori* infection [13]. In particular, miRNA-146a is involved in the regulation of innate immunity and the *H. pylori*-induced inflammatory response, modulating the expression of target genes, such as IL-1 receptor associated kinase 1 (IRAK1) and tumour necrosis factor (TNF) receptor associated factor-6 (TRAF6). miRNA-155 is a crucial regulator of innate immunity and is expressed in a wide variety of immune cells during the inflammatory response, including macrophages, dendritic cells and several types of lymphocytes. During an *H. pylori* infection, miRNA-155 is transcriptionally induced in macrophages in response to Toll-like receptor (TLR) ligands or to TNF-alpha exposure in a manner that is dependent on transcription factors such as activator protein-1 (AP-1) and nuclear factor (NF-κB). However, no studies have described miRNA expression in the gastric mucosa of children in the context of *H. pylori* infections and the development of gastritis or during an infection in the model animal *Meriones unguiculatus*. Thus, in this study, we analysed the differential expression of miRNAs associated with *H. pylori* infection in paediatric and adult patients as well as in the *M. unguiculatus* animal model.

## Methods

### Patients

Selected gastric lesions from paraffin-embedded biopsy files at the Hospital Infantil de México Federico Gómez (HIMFG) Pathology Department were studied and distributed as follows: 16 children diagnosed with *H. pylori*-induced gastritis and 9 children with gastritis without *H. pylori*, all of whom were admitted to the Department of Gastroenterology and Nutrition of the HIMFG. In addition, 37 adult patients from the Manuel Gea González General Hospital and the Gabriel Mancera Hospital were included, 32 of whom were infected with *H. pylori* (13 patients diagnosed with follicular gastritis and 19 with metaplasia) and 5 patients had gastritis without *H. pylori*.

### *H. pylori* infection in *M. unguiculatus*

Twenty five 8-week-old *M. unguiculatus* males (Hsd:MON, Harlan Teklad, WI, USA) that weighted 40 g and were free of specific pathogens were used. The animals were obtained from the animal house of the Hospital Infantil de México Federico Gómez, México City. The Mongolian gerbils were maintained in plastic metabolic cages to prevent coprophagy under the same previously described conditions [14]. Free access to a standard diet (special rodent food; Harlan Teklad, WI, USA) and sterilized tap water were provided. The animals were euthanized by injection of a high dose of chemical anaesthetics (pentobarbital).

Animal care was performed in accordance with national and institutional policies (NOM-062-ZOO-1999) and the Guide for the Care and Use of Laboratory Animals for Animal Health and Well-being [15]. The Ethics, Biosafety and Scientific committees at the Health Institute approved the experiment (HIM/2011/080- SSA 1005).

Each group of gerbils included five animals, ensuring that three of them would present *H. pylori* infection ([16]). Gerbils were inoculated intragastrically with 500 mL NaHCO_3_ (0.2 M) and again 1 h later with a bacterial suspension of *H. pylori* (6 × 10^8^ colony forming units (CFU)/mL) every day for one week. The gerbils were fasted 18 h prior to the first inoculation until the end of the fifth inoculation. The *H. pylori* strains used in this study included the reference strain 26,695, and control animals received saline alone. The gerbils were euthanized at 0, 3, 6, 12 and 18 months by cervical dislocation under anaesthesia to harvest the stomachs. The stomach was dissected along the greater curvature and washed with phosphate-buffered saline (0.01 M PBS, pH 7.4). For each animal, one of the sections was fixed in 4% formaldehyde and embedded in paraffin blocks for subsequent histological analysis [14, 16].

### Histopathological analysis of injuries

Gastric biopsies obtained from paediatric patients, adults and *M. unguiculatus* animals were paraffin-embedded, and histopathological sections were subsequently created. The sections were stained using a Giemsa and haematoxylin-eosin technique and were independently observed and evaluated by an experienced pathologist. Histopathological examination was performed to determine the degree of damage based on the updated Sydney system [17–19]. The parameters evaluated included *H. pylori* positivity, histological grade of neutrophil infiltration (activity), infiltration of mononuclear cells (chronic inflammation), glandular atrophy and intestinal metaplasia. These parameters were scored with ordinal values of 0, 1, 2, and 3, corresponding to null, mild, moderate and severe, respectively.

### Identification of *H. pylori* by detection of the *glmM* gene by PCR

Paraffin-embedded biopsies were sectioned into 10-μm-thick slices, two of which were deposited into 1.5-ml microcentrifuge tubes and dewaxed via immersion in xylene at 50 °C, followed by absolute and 96% ethanol. Genomic DNA was extracted from cultured *H. pylori* using a Wizard Genomic DNA Purification Kit (Promega, Madison, WI, USA,) following the manufacturer’s instructions. DNA was quantified using an Epoch Microplate Spectrophotometer (BioTek, Vermont, USA), and DNA integrity was evaluated by electrophoresis in 1% agarose gels. *H. pylori* was identified by the presence of the *glmM* gene [20]. Amplification was performed in a reaction volume of 25 μL of Master Mix (Promega) containing 100 ng of bacterial DNA, 2.5 mM MgCl2, 10 mM dNTPs, 2 U of Taq DNA polymerase, 20 pmol of each primer and nuclease-free water in a Thermo Hybaid thermal cycler (PCR Express, CA, USA). The PCR products were separated by electrophoresis in 1% agarose gels at 80 V, followed by staining with ethidium bromide and imaging under UV illumination (ChemiDoc transilluminator, BIO-RAD, USA). DNA from *H. pylori* ATCC strain 43,504 was included as a positive control and human DNA was used as a negative control.

### Analysis of miRNA-146a and miRNA-155 expression by quantitative real-time polymerase chain reaction (qRT-PCR)

Paraffin-embedded biopsies were sectioned into 10-μm-thick slices, two of which were deposited into 1.5-ml microcentrifuge tubes and dewaxed via immersion in xylene at 50 °C, followed by absolute and 96% ethanol. Total RNA was extracted using TRIzol® Reagent (Ambion RNA by Life Technologies; Carlsbad, California, USA), followed by a phenol:chloroform extraction. The quality and amount of the resulting RNA was assessed using a NanoDrop spectrophotometer (The Epoch™ Multi-Volume Spectrophotometer System, WI. USA) at 260 and 280 nm, respectively.

The expression of human and mouse miRNA-146a and miRNA-155 as well as two nucleolar RNAs (snoRNAs), RNU6 (present in humans and mice) and RNU48 (humans only), was measured by qRT-PCR. cDNA was synthesized from total RNA using TaqMan miRNA primers and a TaqMan® MicroRNA Reverse Transcription Kit from Applied Biosystems (Foster City, California, USA). For qRT-PCR, the pre-designed probes included with the TaqMan MicroRNA Life Technologies Assay Kit were used, which included primers and a TaqMan hybridization probe from Applied Biosystems (ID 000468, ID 002623, ID 001973 and ID 001006; ID 000468 and ID 002571 were mouse-specific), and the TaqMan® Universal Master Mix II was used with uracil-N-glycosylase (UNG) 2× (Applied Biosystems Foster City, California, USA). Real-time PCR amplification was performed in triplicate under the following conditions: a 2 min incubation at 50 °C (for UNG activation) was followed by a 10 min incubation at 95 °C (for polymerase activation), after which 40 cycles of denaturation at 95 °C for 15 s and alignment and extension at 60 °C for 1 min using a Stratagene Mx3005p qPCR System and MxPro-Mx3005p software (Santa Clara, Calif., USA). The snoRNAs RNU6 and RNU48 were used as endogenous controls to normalize the data. The relative expression of the assayed miRNAs was calculated using the comparative 2-ΔCt method established by Pfaffl et al. (2004) [21].

### Statistical analysis

Data analysis was performed using GraphPad Prism 6.0 (San Diego, CA, USA). ANOVA (Kruskal-Wallis) analyses of variance were used to analyse the statistical significance for unpaired groups, and multiple groups comparisons were performed using the Two-sample Wilcoxon rank-sum (Mann-Whitney U) test [Differences between groups of children with *H. pylori*-infected gastritis vs. children with uninfected gastritis and adults patients (with follicular gastritis and metaplasia) vs. adults with uninfected gastritis]. Differences with a *p* value of < 0.005 were considered significant. For animal model analyses, comparisons were made between the *M. unguiculatus* groups infected with *H. pylori* for different durations vs. the control *M. unguiculatus* group using a one-way ANOVA test, and differences were considered significant when *p* < 0.005. Comparisons were made between *M. unguiculatus* groups at different stages of infection (0, 3, 6, 12 and 18 months) using Student’s t-test. Differences with a *p* value of < 0.005 were considered significant.

## Results

Sixty-two patients were included in this study. Among the 25 paediatric patients (16 males and 9 females), the minimum age was 2 years, the maximum age was 17 years, and the median age was 8 years and 8 months. Among the adults patients [20 males and 17 females], the minimum age was 24 years, the maximum age was 83 years, and the median age was 52 years and 2 months.

### Histopathological analysis

*H. pylori* was identified in all infected groups by endpoint PCR. The results presented in Table 1 show the histopathological characteristics of paediatric patient biopsies based on the updated Sydney system. Of 25 paediatric patients with gastritis, 55.5% (5/9) of *H. pylori*-negative infected children exhibited chronic gastritis, 22.25% (2/9) had chronic follicular gastritis, and 22.25% (2/9) had reactive gastropathy with intestinal metaplasia. In children testing positive for *H. pylori* infection, 62.5% (10/16) had chronic gastritis and 25% (4/16) had chronic follicular gastritis, with two of these cases (12.5% [2/16]) exhibiting reactive gastropathy.

**Table 1:** Histological characteristics of gastric lesions in children with gastritis without *H. pylori* (negative) and children with gastritis who were infected with *H. pylori* (positive) based on the updated Sydney system

Gastritis	Infection with *H. pylori*	N = 25	Activity	Atrophy	Metaplasia
Chronic	Negative	5 (55.5%)	1	L	N
Follicular chronic	Negative	2 (22.25%)	0	N	N
Reactive gastropathy	Negative	2 (22.25%)	0	N	Intestinal
Chronic	Positive	10 (62.5%)			
		(4)	1	N	N
		(2)	1	L	
		(2)	2	L	
		(2)	2	M	
Follicular chronic	Positive	4 (25%)			
		(2)	0	N	N
		(1)	0	L	
		(1)	1	L	
Reactive gastropathy	Positive	2 (12.5%)	1	N	N

Activity: 0 (null); 1 (mild); 2 (Moderate); 3(severe)

Atrophy: *N* Negative, *L* Light, *M* Moderate

**Fig. 1:**
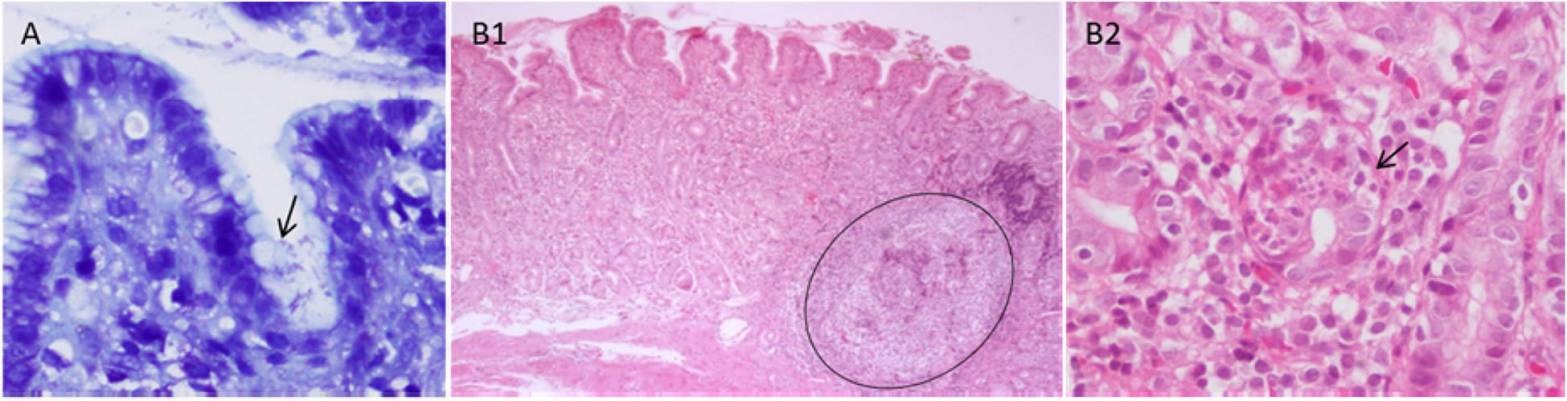
**a** *H. pylori* bacilli infecting the mucosal surface of an antral gland, marked with an arrow. Giemsa stain (60×). **b1**, **b2** Chronic follicular gastritis with moderate atrophy of the gastric mucosa, moderate activity, the absence of metaplasia, and associated with *H. pylori*. Chronic follicular gastritis was characterized by the presence of intraepithelial polymorph nuclear inflammatory infiltration, expansion of the lamina propria at the expense of a dense mixed infiltrate consisting of both polymorph nuclear and mononuclear lymphocytes and plasma cells, and the formation of lymphoid follicles with germinal centres (arrows and circles) (haematoxylin-eosin), observed at 10× and 60× magnification

Some of the histopathological findings observed in the gastric lesions of children infected with *H. pylori*, shown in Fig. 1, indicated the presence of *H. pylori* bacilli in the antral gastric mucosa as well as mucosal damage caused by the presence of inflammatory cells, which invade the gastric glands and promote the loss of their morphology and function.

In adult patients testing positive for *H. pylori* infection, chronic follicular gastritis was observed in 13 cases and reactive gastropathy with metaplasia in 19 cases, while adult patients without infection exhibited follicular gastritis.

### Expression of miRNA-146a and miRNA-155 in gastric lesions of children and adults infected with *H. pylori*

**Fig. 2:**
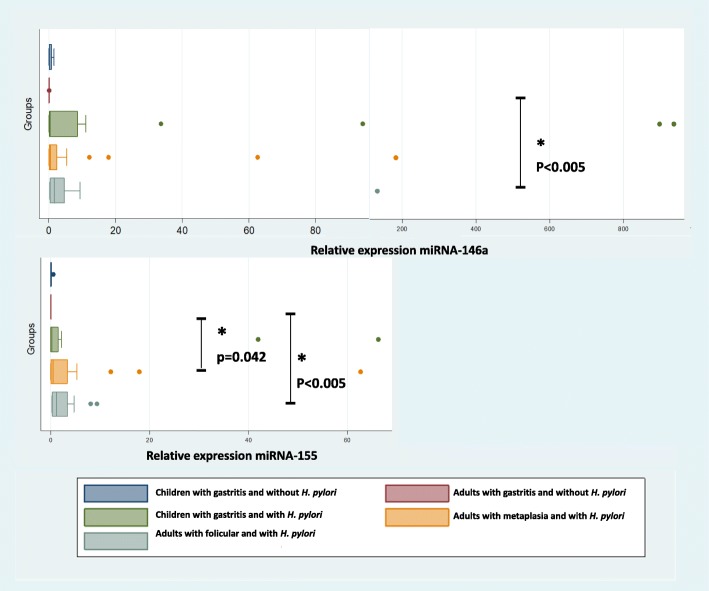
miRNA expression in human gastric tissue. Expression of miRNA-146a and miRNA-155 was determined in the gastric lesions of *H. pylori* children and adults as well as *H. pylori* children and adults with gastritis by performing qRT-PCR (**a**, **b**). miRNA levels were normalized to RNU48 and RNU6 levels. The median, maximum and minimum values ​​of three independent experiments are shown. The data are presented as box-whisker plots (box, 25–50%, whiskers, 5–95%, and line, median). ** *p* < 0.005

miRNA-146a and miRNA-155 were differentially expressed in the gastric lesions of children and adults infected with *H. pylori* (Fig. 2). The detailed of expression of miRNA-146a and miRNA-155 and data patients (age and sex) are listed in Additional file 1: Table S1.

The relative expression levels of miRNA-146a and miRNA-155 in paediatric patients infected with *H. pylori* were 247.6- and 79.4-fold greater than those observed in uninfected patients, respectively. Increased miRNA expression was observed with respect to gastritis activity. For example, in non-active gastritis, we observed a 1.53-fold increase in the relative expression of miRNA-146a, whereas in patients exhibiting severe gastritis, miRNA-146a expression increased 935.7-fold. Similar results were observed for miRNA-155, which was increased 0.12-fold in non-active gastritis and up to 66.25-fold in severe gastritis. Although the average miRNA-146 and miRNA-155 expression increased greatly in the mucosa of *H. pylori*-positive paediatric patients with gastritis, these differences were not significant (*p* = 0.12 and 0.07, respectively).

Adult patients infected with *H. pylori* exhibited increased relative expression levels of miRNA-146a and miRNA-155 (21.79- and 62-fold, respectively) in follicular gastritis compared to that observed in uninfected adult patients. In addition, in patients infected with metaplasia, 7.5- and 124.5-fold increases in the relative expression levels of miRNA-146a and miRNA-155 were observed compared to those seen in uninfected patients, respectively. The average increase in miRNA-146 and miRNA-155 expression was significant for follicular gastritis (*p* < 0.005) and metaplasia (p = 0. 93 and 0.042, respectively).

### Histopathological analysis of gastric lesions in *M. unguiculatus*

Table 2 shows the histopathological results for the gastric lesions observed in *M. unguiculatus* during different stages of *H. pylori* infection. After 3 months of infection, a mild development of gastritis was observed, which increased to moderate and severe activity with atrophy over time (6, 12 and 18 months).

**Table 2:** Histological characteristics of gastric lesions in *M. unguiculatus* infected with *H. pylori* based on the updated Sydney System

Stage of infection	Length of Infection (months)	Activity	Atrophy	Metaplasia
Control	0	0	0	0
Colonization	3	1	N	N
Persistence	6	2	L	N
Chronicity	12	3	M	N
Chronicity	18	3	M	L

Activity: 0 (null); 1 (mild); 2 (Moderate); 3(severe)

*N* Negative, *L* Light, *M* Moderate

### Differential expression of miRNA-146a and miRNA-155 in gastric lesions of *M. unguiculatus* infected with *H. pylori*

**Fig. 3:**
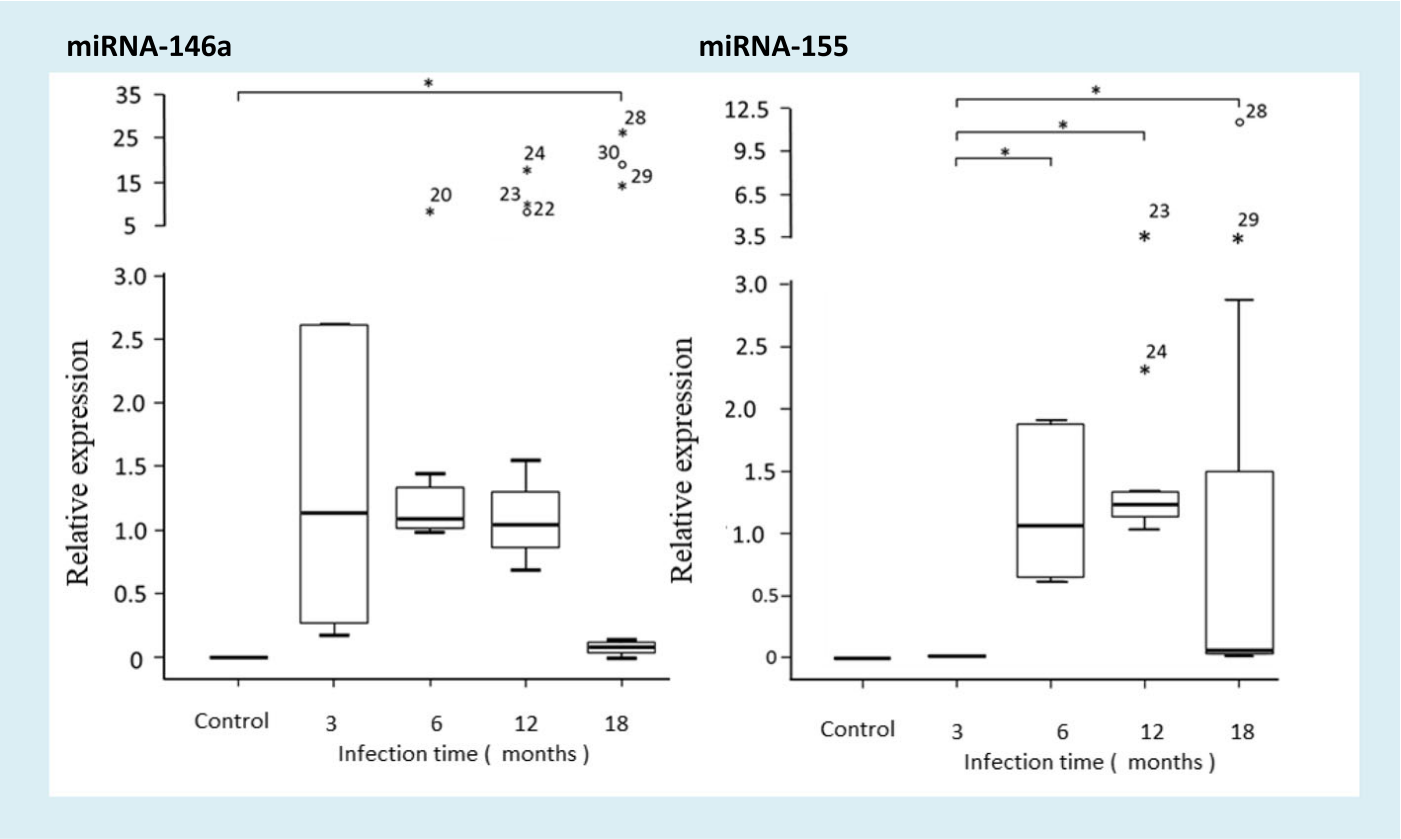
miRNA expression in gastric lesions of *M. unguiculatus*. Expression of miRNA-146a and miRNA-155 was determined in the gastric lesions of *M. unguiculatus* infected with *H. pylori* 26,695 for 3, 6, 12 and 18 months and in *H. pylori* (−) *M. unguiculatus* by performing qRT-PCR. miRNA levels were normalized to RNU6 levels. The median, maximum and minimum values ​​of three independent experiments are shown. The data are presented as box-whisker plots

In gastric lesions of *M. unguiculatus* infected with *H. pylori* 26,695, the expression of miRNA-146 increased throughout the duration of the infection. However, a significant difference was only observed at 18 months when compared to the uninfected group. In contrast, miRNA-155 expression levels were detected after 6 months of infection. miRNA-146a expression levels increased over time by 1.32-, 1.92-, 6.83- and 10.54-fold (*p* < 0.005) at 3, 6, 12 and 18 months, respectively, and by 1.19-, 1.79- and 3.04-fold, respectively (*p* < 0.005), when compared to those in the uninfected group. miRNA-155 expression levels increased slightly compared to those observed in the uninfected group (Fig. 3).

## Discussion

*H. pylori* infection is acquired during childhood and persists into adulthood. Infected individuals may develop gastritis, and various studies have demonstrated a relationship between gastritis and altered miRNA expression, specifically altered expression of various miRNAs in the gastric lesions of adult patients infected with *H. pylori* [8, 22, 23]. However, there have been no reports of miRNA expression levels in children infected with this bacterium.

In this first study conducted in paediatric patients with *H. pylori*-infected gastritis, the results showed that the relative expression levels of miRNA-146a and miRNA-155 in paediatric patients infected with *H. pylori* were folds-greater when compared to those observed in uninfected patients. However, the differences were not significant. The above results occurred because during *H. pylori* infection, the gastric epithelial cells recognize the TLRs of the bacteria and may then activate a series of inflammation-related signalling pathways. For example, *H. pylori-* bacterial lipopolysaccharide (LPS), a cell wall component, is recognized primarily by TLR4 [24]. TLR4 can activate the myeloid differentiation primary-response gene 88 (MyD88), leading to the translocation of NF-κB into the nucleus, which subsequently activates the expression of genes related to the inflammatory process. Similarly, several TLR ligands have been shown to increase miR-155 expression via MyD88 [25]. However, studies of *H. pylori*-infected children have not indicated the occurrence of changes in the expression of natural immunity receptors such as TLR2 and TLR4 as well as the adapter protein MyD88, unlike in adult patients [26].

In children infected with *H. pylori*, the host response is characterized by an increase in lymphocytic infiltration in the gastric mucosa and a high expression of anti-inflammatory mediators. Such a response is related to the tolerance of macrophages and dendritic cells, which contributes to the formation of different suppressor T cells, including Tregs, Tr1 and Th3 cells [27]. In contrast, miRNAs are involved in the downregulation of pro-inflammatory cytokines through the attenuation of NF-κB [28], including miRNA-146a-mediated immunological tolerance and T-cell differentiation mediated by miRNA-155 [29]. This potentially explains the gradually increased levels of miRNA expression observed in infected paediatric patients. In particular, in three patients overexpressing miRNA-146a and miRNA-155, moderate to severe active gastritis was observed, and the presence of parasites was ruled out. This suggests that the miRNA overexpression in these patients is closely related to lymphocytic infiltration.

The results obtained for miRNA-146a and miRNA-155 expression in the tissue of adults with follicular gastritis agrees with the findings of Xiao et al. (2009), Liu et al. (2010) and Lario et al. (2012), who reported that miRNA expression increased in the lesions of patients with infected gastritis compared to those with normal gastric mucosa (without gastritis). Specifically, for miRNA-146a in tissue infected with *H. pylori* [22, 23, 30]. In addition, there have been reports of a correlation between miRNA-146a and miRNA-155 expression, which are overexpressed in gastric lesions with *H. pylori*. TLRs play an important role in maintaining gastric mucosal homeostasis, and TLRs signal through the adaptor molecule MYD88 after *H. pylori* recognition, which activates downstream factors to coordinate the upregulation of several functionally distinct gene subsets. In particular, miRNA-146a and miRNA-155 are regulated by TLR in different cell types, some of which fine-tune the control of TLR signalling components [24, 25].

The expression of miRNA-146a is altered in some types of tumours [31], particularly in gastric cancer, where it acts as a tumour suppressor by inhibiting cell migration and invasion [32]. In addition, miRNA-155 acts as an oncogenic miRNA (oncomiR), and the expression of miRNA-155 is increased in different types of leukaemia, lymphomas and solid tumours of various origins. The expression levels of miRNA-155 are higher than those of miRNA-146a in these diseases, whereas in this study, we observed that miRNA-146a and miRNA-155 are not expressed in patients with metaplasia, which is considered a preneoplastic lesion. miRNAs have different levels of expression in gastric cancer tissues that perform different functions. Li et al. (2014), observed significantly lower expression levels of miRNA-155 in gastric carcinomas than those of corresponding non-tumour gastric tissues [33]. The negative regulation of miRNA-155 accelerates cell growth and directs the invasion of c-myc into human gastric carcinoma cells [34]. c-Myc serves as a pro-oncogene that is closely related to tumourigenesis and sustained tumour growth [35]. Therefore, miRNA-155 can function as a gastric tumour suppressor during the development of gastric carcinoma. Thus, the regulation of miRNA-155 during gastric tumourigenesis is a complicated and poorly understood.

Different animal models have been developed to study *H. pylori* infection, including rats and different strains of mice that have been successfully infected with this bacterium. However, the study of gastric pathologies induced by *H. pylori* has been difficult to describe because the available models are able to resolve infection and do not develop pathology. Hirayama et al. (1996) reported another rodent infection model, *M. unguiculatus*, commonly known as the Mongolian gerbil [36]. In this model, bacteria are detected for long periods of time and produce lesions, such as active chronic gastritis, peptic ulcers and intestinal metaplasia, similar to those generated in humans. This model is also capable of developing gastric carcinogenicity using a carcinogenic agent, such as N-methyl-N-nitrosourea in conjunction with *H. pylori*. Despite the various advantages of this model with respect to infection, there have been no reports of the expression of *H. pylori*-induced miRNAs in *M. unguiculatus*. Thus, this is the first report to describe the expression of miRNAs, specifically miRNA-146a and miRNA-155, in *M. unguiculatus* infected with this bacterium at different time points. In addition, the results of this study showed that these miRNAs are expressed and continue to increase throughout the infection. However, our model had limitations, and we determined that miRNA expression levels in *M. unguiculatus* are much lower relative to those observed in humans This may be related to the chronicity of the infection, because the period of infection in *M. unguiculatus* is short (3 to 18 months) compared to the course of chronic human infections, which occur over much longer periods of time, spanning approximately 20 years from the acquisition of bacteria to the establishment of disease.

Previous studies performed by our group have demonstrated that infection with *H. pylori* strain 26,695 in *M. unguiculatus* is subject to changes over time due to the loss or acquisition of genetic material via genetic recombination events [16]. We also suggest that the bacterium undergoes an adaptation process in the gastric mucosa of the animal model, because important intragenic changes and few histological changes are observed in the gastric mucosa. This observation may be related to the slight increase in genetic changes observed throughout the infectious process. Philpott et al. (2002) reported that some strains of *H. pylori* can adapt in mice [37]. Such adaptation prevents the bacteria from causing damage to the mucosa due to a significant reduction in the inflammatory process in the gastric mucosa, indicated by the inability to induce the secretion of pro-inflammatory cytokines.

Several studies have suggested that chronic gastritis is associated with the alteration of miRNA-155 and reported a gradual increase trend of miRNA-155 expression in preneoplastic gastric mucosa, indicating that miRNA-155 is essential to the persistent inflammation of gastric mucosa [38-40]. Similarly, in chronic gastritis it was observed that overexpression of miRNA-146a could significantly decrease the activity of the nuclear factor-kappa B (NF-*κ*B) pathway, suggesting that miRNA-146a may play a crucial role in a negative feedback loop to modulate gastric mucosal inflammation [22].

## Conclusion

In this study, we showed that the increased expression of miRNA-146a and miRNA-155 is associated with the duration and outcome (follicular gastritis or metaplasia) of *H. pylori* infection. In humans, *H. pylori* induces a persistent chronic infection, during which the gastric epithelial cells recognize the TLRs of the bacteria and then may activate a series of inflammation-related signalling pathways., In addition, the loss or acquisition of genes by this bacterium facilitate its survival and persistence within the host. Therefore, it is important to consider this during the *H. pylori* infectious process in paediatric patients, as important changes are taking place during this developmental period. In addition, the differential expression of miRNAs, specifically miRNA-146a and miRNA-155, which we demonstrated are associated with gastritis and undergo important changes in expression in both paediatric patients and adults, is indicative of the chronicity of infection and disease severity.

## Additional file

Additional file 1: **Table S1.** Expression of miRNA-146a and miRNA-155 in the gastric lesions of children and adults infected with and without *H. pylori*. miRNA levels were normalized to RNU48 and RNU6 levels. (XLSX 19 kb)

## Abbreviations

AP-1: Activator protein-1
CFU: Colony forming units
IFNγ: Interferon gamma
IRAK1: IL-1 receptor associated kinase 1
miRNA: microRNA
MyD88: Myeloid differentiation primary response 88
NF-κB: Nuclear factor
oncomiR: Oncogenic miRNA
qRT-PCR: Quantitative real-time polymerase chain reaction
RNU48: Nucleolar RNAs (humans only)
RNU6: Nucleolar RNAs (present in humans and mice)
snoRNAs: Small nucleolar RNAs
TLR: Toll-like receptor
TNF: Tumour Necrosis factor
TRAF6: Receptor associated factor-6
TREM1: Triggering receptor expressed on myeloid cells 1
UNG: Uracil-N-glycosylase
